# Shaping complex microwave fields in reverberating media with binary tunable metasurfaces

**DOI:** 10.1038/srep06693

**Published:** 2014-10-21

**Authors:** Nadège Kaina, Matthieu Dupré, Geoffroy Lerosey, Mathias Fink

**Affiliations:** 1 Institut Langevin, ESPCI ParisTech and CNRS UMR 7587, 1 rue Jussieu, 75005 Paris, France

## Abstract

In this article we propose to use electronically tunable metasurfaces as spatial microwave modulators. We demonstrate that like spatial light modulators, which have been recently proved to be ideal tools for controlling light propagation through multiple scattering media, spatial microwave modulators can efficiently shape in a passive way complex existing microwave fields in reverberating environments with a non-coherent energy feedback. Unlike in free space, we establish that a binary-only phase state tunable metasurface allows a very good control over the waves, owing to the random nature of the electromagnetic fields in these complex media. We prove in an everyday reverberating medium, that is, a typical office room, that a small spatial microwave modulator placed on the walls can passively increase the wireless transmission between two antennas by an order of magnitude, or on the contrary completely cancel it. Interestingly and contrary to free space, we show that this results in an isotropic shaped microwave field around the receiving antenna, which we attribute again to the reverberant nature of the propagation medium. We expect that spatial microwave modulators will be interesting tools for fundamental physics and will have applications in the field of wireless communications.

Controlling wave propagation for focusing or imaging applications is of significant interest in many domains which range from medical imaging and therapy to wireless communications or nanolithography, to name a few. Controlling waves in homogeneous media such as air is relatively easy and has long been realized using lenses in optics^1^. These apply a path difference to every ray going from one point to another, in order to allow for constructive interferences of waves at a given location of space. Similarly, reflectarrays have been proposed in the microwave domain in order to beam waves coming from a horn antenna^2^,^3^,^4^,^5^,^6^. Again, here, the idea is to reflect the waves off a planar matrix of resonators of different sizes, which present at a given frequency a distribution of resonance frequency and hence all reflect the incoming wave with a phase shift related to their dimensions^2^,^7^. Reflectarrays are widely used for satellite communications, and are the ancestors of the concept of metasurface, which is extensively studied nowadays for free space applications in microwave, acoustics or optics^8^,^9^,^10^,^11^,^12^,^13^,^14^.

When it comes to controlling the propagation of waves in heterogeneous and complex media, however, the problem is more complex since scattering and diffraction can turn a plane wave into a completely random wave field, namely, a speckle^15^. Heterogeneous and complex media include multiple scattering and reverberating ones, or media which are both, and they were thought until a few years back to be of no use for wave physicists, since they scramble waves in an extremely complex way. Yet it was shown two decades ago that these seemingly useless media can be taken advantage of for focusing or imaging purposes through the concept of time reversal. Time reversal, the broadband equivalent of phase conjugation, permits to harness scattering and reverberation in order to focus waves far below the Rayleigh limit which is given by the source aperture in free space^16^,^17^,^18^. It was even shown that time reversal, associated with locally resonant metamaterials, can potentially focus waves from the far field way below the diffraction limit^19^,^20^,^21^.

Even more recently, pioneering experiments have been realized using spatial light modulators (SLMs) in optics that showed the possibility to focus light through or in multiple scattering media^22^,^23^,^24^,^25^,^26^, for instance for imaging applications^27^,^28^. SLMs consist in matrices of micro-mirrors or liquid crystal cells which impose a physical phase shift to the portion of light they reflect. In the seminal work, a simple incoherent energy based feedback was used alongside an optimization algorithm to focus light on a single speckle grain through a thick layer of commercial paint^29^. The idea, in this work and the following ones, is to control the phase and/or amplitude of independent speckle grains at the input of a multiple scattering medium, in order to add them in phase at its output, thus obtaining a focal spot whose intensity varies linearly with the number of controlled grains^30^.

In this article, we apply the same idea to the field of microwaves using a homemade electronically tunable metasurface that we propose to use as a spatial microwave modulator. Yet we perform our demonstration inside a reverberant medium rather than through a multiple scattering one, since it is a more common kind of medium at these frequencies. To that aim, we first design the spatial microwave modulator (SMM), or in other words the tunable metasurface. We prove experimentally that owing to the speckle-like nature of wave fields existing in complex reverberating media, a binary only phase modulation permits a very good shaping of waves that we quantify later on. Specifically, we demonstrate in a typical office room that the spatial microwave modulator can be placed on the walls in order to passively shape the wave field and improve by an order of magnitude the wireless transmission between two antennas, or on the contrary to cancel completely this transmission. Then we underline and explain that while reflectarrays and metasurfaces usually create elongated foci in the direction of propagation, in reverberating media isotropic foci or null can be obtained. Finally we discuss the potential applications of the approach, which could be found in fundamental physics as well as in the future for greener wireless communications.

## Results

### Design and characterization of the spatial microwave modulator

We start by creating the tunable metasurface, which will cover part of the walls of a typical office room. To do so, we first design its unit cell, namely, the planar resonator that will reflect the waves with a controllable phase shift. We choose to work with resonators that are rectangular patches sitting on a ground plane^31^ since our metasurface will anyway be placed on walls, which are ground planes albeit poor ones. For the sake of simplicity and rapid convergence of the optimizations, we opt for the simplest case of a binary phase modulation, that is, a two states resonator that reflects the waves either positively or negatively (see Supporting Information). As in binary amplitude modulation realized in optics^32^ and contrary to free space beamforming it will be very efficient since we deal with wave fields resulting from reverberation in a complex medium, that is, fields which vary drastically on the scale of half a wavelength. To do so, the most trivial choice is a resonator that presents a resonance frequency *f_ref_* which can be shifted using an electronic circuit. If *f_ref_* is set such that it corresponds to the working frequency *f_0_*, the resonant unit cell reflects the waves at this frequency with a π phase shift as compared to the bare ground plane, this phase shift being due to the resonance itself. Now when *f_ref_* is shifted away from *f_0_*, the unit cell is non-resonant at this frequency, and the ground plane reflects the waves with a 0 relative phase shift. We stress here that we define the phase shift of the reflected waves relatively to that of the non-resonant unit cell as in^31^, since it is general and can be applied to any kind of unit cell.

**Figure 1:**
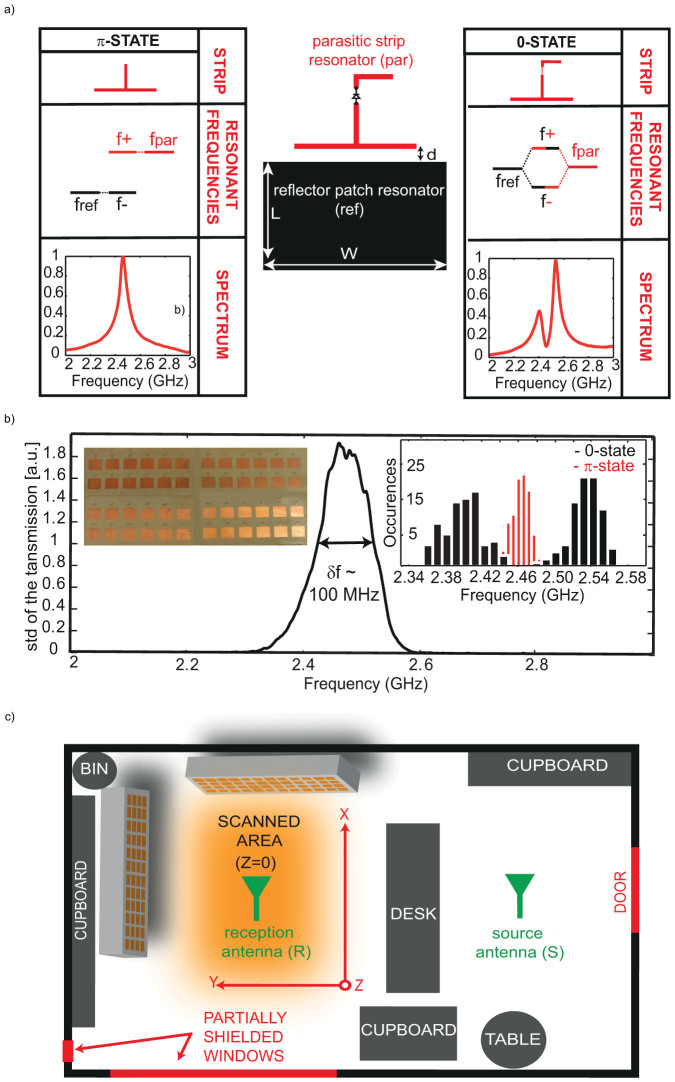
(a) the resonant unit cell (middle) and its two operating states (left-right). (b) Standard deviation of the transmission between antennas (S) and (R) for 11000 random configurations and 10 positions of (S), one panel (out of 2) of the metasurface (inset left) and the distribution of resonance frequencies of the 102 resonators for both states measured with near field probes (inset right). (c) Schematic view of the setup and its surrounding environment.

In fact we opt for a slightly more complicated design than a bare resonating cell which consists of two strongly coupled or hybridized resonators^31^ as described in Fig. 1(a) and detailed in the Methods section and in the Supporting Information. The first resonator is called the reflector patch resonator. It is again a patch sitting on a ground plane, polarized along its short axis, and whose resonance frequency *f_ref_* is set to *f_0_*. The second resonator, the parasitic one, is a stripline sitting on the ground plane and near field coupled to the reflector. Its resonance frequency *f_par_* can be electronically tuned from *f_0_* to a higher frequency *f_1_* using a diode. When *f_par_* is set to *f_1_*, the reflector resonance frequency *f_ref_* is unchanged and it reflects the waves with a π phase shift as compared to the bare ground plane (π-state). In contrast when the *f_par_* is shifted to *f_0_*, the two resonators hybridize and a dimer presenting two resonant frequencies *f_−_* and *f_+_* respectively below and above *f_0_* is created. In this state, at *f_0_*, the dimer is again transparent and the waves are reflected by the ground plane with a 0 relative phase shift (0-state). This design presents notable advantages: the reflection properties of the unit cell are insensitive to both the losses and impedance variations of the electronic components and to the soldering which are placed on the parasitic resonator only (see Supporting Information).

We fabricated a 0.4 m^2^ SMM consisting of 102 controllable electromagnetic reflectors (half of them pictured in the inset of Fig. 1(b)), spaced by half a wavelength at the working frequency *f_0_* = 2.47 GHz, that is, 6 cm; this tunable metasurface, which is 1.5 mm thin, will be our SMM. A simplified experimental setup is shown in Fig. 1(c). We control the 102 elements metasurface using two Arduino 54 channels digital controllers and an Agilent network analyzer is used to measure the transmission between the source (S) and receiver (R) antennas the latter being either a regular monopole antenna or an electro-optic probe when spatial scanning is realized. We use commercial monopole Wi-Fi antennas polarized along the same axis as the metasurface resonators. Antenna (S) is placed far away and out of sight of both (R) and the metasurface in a furnished and hence scattering 3 × 3 × 4 m^3^ office room. This antenna thereby creates in the whole room and notably on the SMMs a random microwave field. Antenna (R) is placed one meter away from the metasurface (see Fig. 1(c)).

We first characterize the designed SMM. To do so, we measure the resonance frequency of each unit cell out of the 102 elements of the array in the 0 and π states, using homemade near field probes. We plot in the inset of Fig. 1(b) the resonance frequencies histogram. It gives us the following information: the π-state distribution is relatively narrow and centered on *f_0_*, while the 0-state distribution is somewhat broader due to the electronic components but still does not overlap the π-state one. We also evaluate the bandwidth of the metasurface by displaying 11000 random configurations (out of 2^102^ of the 102 binary resonators array) and measuring the standard deviation of the transmission between (S) and (R). We do so for 10 different positions of (S) to average over disorder (see Methods). This smoothes the spectral variation of the transmission that is due to the room and gives an estimate of the efficiency of the SMM as a function of frequency. We measure a bandwidth of 100 MHz which can be attributed to the bandwidth of each resonator and their dimension distribution due to fabrication uncertainties.

In the experiments, we use this SMM to passively shape the existing multiply reverberated waves in the office room, either to maximize the transmission between antennas (R) and (S) or on the contrary to completely cancel it. Here the source of microwaves is hence the network analyzer connected to antenna (S), which creates a random wave field. Yet we emphasize that since the feedback mechanism used in the algorithms is not coherent, optimizing this random electromagnetic field or any other existing one is strictly equivalent.

### Using the SMMs to improve the transmission between two antennas

**Figure 2:**
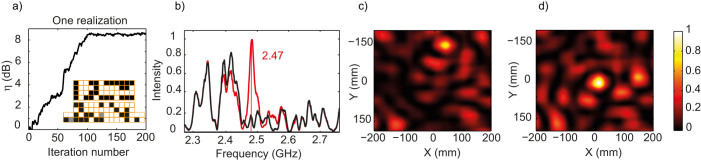
(a) Intensity enhancement over iteration number for one optimization. (b) Initial (resp. final) intensity spectrum in solid black (resp. red) curve. (c) Initial measured intensity map for one realization of the experiment. (d) Final measured intensity map for one realization of the experiment. (a-inset) Final phase mask with each element in 0-state (black) or π-state (white), the grey ones being broken. All maps and spectra are normalized to the maximum of the intensity after maximization.

We now use an intensity feedback mechanism and the procedure demonstrated in optics^29^ to optimize in a passive way the reflection of the multiply reverberated waves off the metasurface such that they focus on (R). We stress here that the term passive that we employ refers to the fact that the SMM does not generate any additional microwaves in the room but simply reflects more intelligently existing ones. We start with a uniformly reflecting metasurface (all elements in 0-state). Then, we iteratively switch each element of the metasurface to the π-state and we measure each time the intensity received on (R) using the network analyzer. This energy feedback is provided by the computer to the metasurface, that is, if the received energy is higher, the element is kept on the π-state, otherwise it is switched back to the 0-state. Here, we emphasize that the order that is chosen to optimize the pixels is of no importance since all the pixels are independent. This condition is met in our experiment since all cells are separated by a correlation length of the medium (λ/2) and since the room is a low Q-factor medium, preventing the waves from bouncing on several different pixels before dying off, which would include a correlation between them. We perform 30 optimizations to average over disorder (see Methods). For each one we scan the microwave field before and after optimization using a non perturbative electro-optic probe. Fig. 2(a) displays the enhancement η of the intensity obtained during the optimization process, defined as the ratio between intensities measured after and before maximization. Note that it saturates after about 100 iterations, meaning that the optimization is linear versus the number of pixels used, as already proved in optics^29^. Fig. 2(b)-(c)-(d) show the corresponding intensity spectra of the transmission between (S) and (R) and a map of the field intensity around (R) positioned at the (0,0) point, before and after the maximization. Clearly, even though (S) is out of sight of both (R) and the metasurface, the latter can passively focus the multiply reverberated waves onto (R) on a half wavelength wide focal spot. This literally turns the random wave field into a focused one, thereby providing a net gain of 8.5 dB on (R), almost a decade. Here it is very important to note that focusing the waves using the SMM does not create any shadow where the electromagnetic energy is null, as opposed to what would happen in free space. Indeed, since the medium is reverberating, waves still propagate throughout the whole medium as before optimization, the only difference being that they add up in phase at the focal spot. This conclusion is corroborated first by the spectra (Fig. 2 (b)) that indicates that the effect survives over a 30 MHz bandwidth, limited by the correlation frequency of the room. Another clue of the importance of reverberation in the propagation is that the focal spot observed in Fig. 2(d) is isotropic in the map plane, independently of the fact that the SMM is hanged on a specific side of the room. The inset of Fig. 2(a) presents the phase mask obtained at the end of the optimization, which proves that the field on the metasurface is random (see Supporting Information).

**Figure 3:**
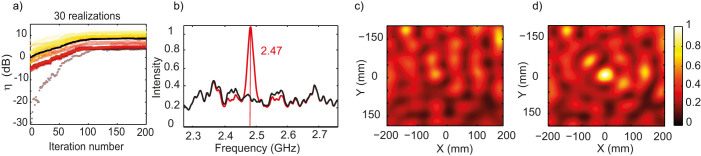
(a) 30 realizations of the maximization plotted in colors, and average in black. (b) Initial (resp. final) intensity spectrum in solid black (resp. red) curve averaged over 30 realizations of the experiment. (c) Initial measured intensity map averaged over 30 realizations of the experiment. (d) Final measured intensity map averaged over 30 realizations of the experiment. All maps and spectra are normalized to the maximum of the intensity after maximization.

In Fig. 3(a)-(b)-(c)-(d), we present the same results averaged over 30 realizations of disorder. For the sake of clarity the data of 3(a) are normalized by the measured average intensity before optimization, while those of 3(b)-(c)-(d) are normalized by the maximum of the average intensity measured after optimization. It shows that the average enhancement obtained is around 8.5 dB, with a minimum of 5 dB, for an intensity which was relatively high before optimization, and a maximum of 35 dB, more than three orders of magnitude, for a very low initial intensity. Spatially, on average, the field is tightly focused around (R); again on an isotropic focal spot half a wavelength wide, consequence of the reverberation. The mean intensity spectrum, which is almost flat before optimization, meaning that the 30 realizations have almost erased the effect of reverberation, clearly displays a peak at 2.47 GHz after maximization, as a signature of the focusing effect. We have also performed experiments with both (R) and (S) out of sight of each other and the metasurface, the three parts being spaced by about 3 meters (see Supporting Information). They prove that even in this worst case scenario ((S) and (R) out of sight of each other and of the SMM) enhancements between 1.5 dB and 6 dB are realizable, with an average of 2.5 dB (Fig. S9). At this point, it is worth noting that the metasurface used is only 0.4 m^2^ as compared to the total wall surface of the 3 × 3 × 4 m^3^ room; results would of course be much better with a larger SMM.

### Cancelling the transmission between two antennas

If multiply reverberated waves can be harnessed to focus onto an antenna, they can also be recycled to cancel the electromagnetic field in a given volume thanks to interferences. To demonstrate this, we use the same optimization procedure as before, albeit with an energy minimization goal. The reception is minimized on antenna (R) which is again placed at position (0,0), and an electro-optic probe is used to scan the field before and after the minimization without perturbing it. We perform 30 measurements in order to average over disorder and, again, the energy feedback is transmitted by the computer to the metasurface during the procedures, the only difference being that the pixel value giving the lowest transmitted energy between (S) and (R) is saved.

**Figure 4:**
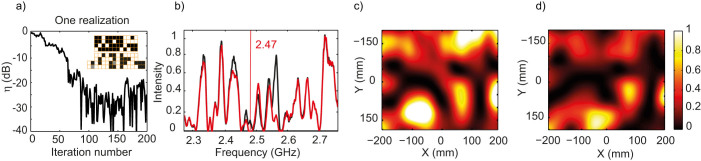
(a) Intensity drop over iteration number. (b) Initial (resp. final) intensity spectrum in solid black (resp. red) curve. (c) Initial measured intensity map for one realization of the experiment. (d) Final measured intensity map for one realization of the experiment. (*a*-inset) Final phase mask with each element in 0-state (black) or π-state (white), the grey ones being broken.

The results for the minimization of a single realization are presented in Fig. 4(a)-(b)-(c)-(d), in a similar manner to the results for the optimization, including the measured intensity spectra and maps before and after the minimization, as well as the diminution of the intensity η (ratio of intensity before and after minimization) as a function of the iteration number. This proves that an optimized random binary phase mask permits (see Supporting Information), as opposed to the bare wall, to decrease the electric field on the receiver (R) by about 25 dB. The spatial scans and intensity spectra further confirm that the field has been cancelled locally around 2.47 GHz.

**Figure 5:**
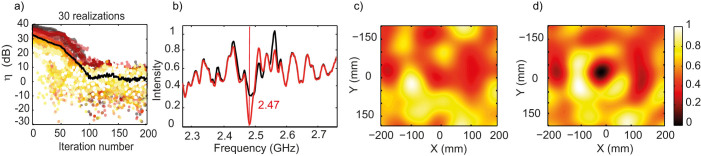
(a) Intensity drop over iteration number for 30 realizations of the experiment, average in black. (b) Averaged initial (resp. final) intensity spectrum in solid black (resp. red) curve. (c) Initial measured intensity map for averaged over 30 realizations of the experiment. (d) Final measured intensity map averaged over 30 realizations of the experiment. All maps and spectra are normalized to the maximum of the intensity before minimization.

The results of the 30 realizations of disorder are displayed in Fig. 5(a)-(b)-(c)-(d). The intensities versus iteration number in Fig. 5(a) have been normalized by the averaged intensity after minimization, that is, the experimental noise level. As for the maps and spectra in Fig. 5(b)-(c)-(d), they have been normalized to the maximum of the average intensity after minimization. Again, we clearly see that the averaging has been correctly performed since the average field intensity is almost constant on the initial spatial scan, and the averaged initial spectrum almost flat on the considered bandwidth. The 30 measurements prove that the field can indeed be cancelled at the desired location around *f_0_*, onto a volume of about half a wavelength cubed, down to a level which is here limited to our measurement noise to about −28 dB. Evidently, higher initial intensities lead to more drastic η of about −35 dB, while lower initial intensities amount to about 20 dB intensity drops, all realizations converging towards the experimental noise level.

## Discussion

**Figure 6:**
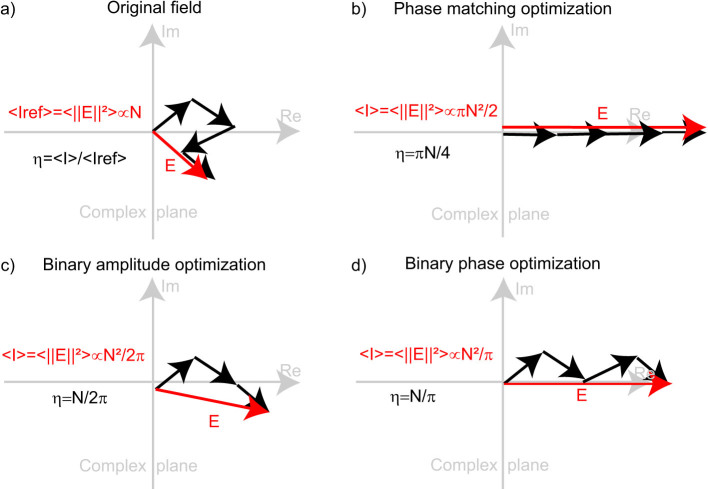
Effect of the binary phase only optimization, seen in the complex plane of the transmission coefficient between two antennas.

First we would like to discuss the idea of using a binary phase modulation for wavefront shaping. Interestingly, we can see that the field in the room (Fig. 2(c)) is a speckle pattern either before the optimization or after (excluding the focal spot). The fact that we cannot see a beam converging to a focal spot after the optimization shows that the focal spot is obtained thanks to the multiply reverberated waves in the room, and that the SMM does not simply behave as a focusing lens. This justifies our approach to use a tunable binary phase metasurfaces. Indeed, a speckle-like field displays very strong spatial variations on distances that are related to the coherence length of the field, which is about half a wavelength here. This means that the electromagnetic field in the complex medium used here changes sign roughly every half wavelength. Hence focusing on a spot requires only an abrupt modification of the phase of the reflected waves, typically a local binary 0 or π phase modulation, contrary to what typically happens for reflectarrays used for beamforming applications, which require a very fine phase tuning of the pixels. Of course, there is a minor loss of the enhancement compared to a complete 2π linear phase optimization. In multiple scattering media, it was shown that with a binary amplitude algorithm^32^ (where the field is transmitted if positive and blocked if negative), the average enhancement of the optimization procedure is roughly N/2π, with N the number of uncorrelated pixels controlled, to be compared to the πN/4 obtained for a perfect phase matching optimization. In our case, instead of cancelling the value of the field on about half the pixels of the SMM element (those for which the field is negative), we simply change the sign of the waves they reflect. This amounts to add only positive contributions for the field after optimization, even though they are not perfectly aligned as in linear phase optimization (Fig. 6). For a random field, we initially have half of the elements that contributes negatively to the intensity, while the other half contributes positively. Hence, after optimization, the enhancement of a binary phase optimization is expected to be twice the one of a binary amplitude optimization as half of the elements now reflect a non-zero amplitude wave.

Next, we would like to discuss the possible outcomes of our approach. First, we believe that it could bring benefits to fundamental physics. Indeed with such SMMs, one can perform wavefront shaping as with SLMs in optics to design a microwave field at will, in order to optimize some specifics modes, be it in a disordered or ordered, chaotic or regular, reverberating or highly multiple scattering medium. The SMM can also find a second application in the field of radio or microwave protection. Indeed, we have shown experimentally that our approach allows concealing the field around one single antenna on a correlation length wide area (~6 cm at 2.4 GHz). In a practical application, a passive microwave receiver could be used to measure the electromagnetic energy and send the feedback to the metasurface, in order to locally cancel an electromagnetic field. Concealing a larger volume would be more challenging but still possible using the Helmholtz-Kirchhoff theorem.

Finally and more importantly, we expect that our approach may bring applications in the field of wireless communications. Indeed, as stated before, indoor environments, especially concrete and metal based constructions are leaky cavities for electromagnetic waves. We proved experimentally that one can use spatial microwave modulators to improve the transmission through specifics channels (modes) of the medium, between a source and one or several receivers by orders of magnitude. One could then use a SMM as a smart wall in an indoor environment between an internet router and a computer or between a cell phone and its base station equivalently, since the necessary feedback is incoherent. The smart phone could for instance use an application which measures in real time the binary error rate of an ongoing communication, and sends a binary feedback based on this estimate to the metasurface. Our approach could hence be already used in poorly covered areas to greatly increase the quality of reception of wireless communications or Wi-Fi systems, but it could also lead to much greener telecommunications in the future. Indeed this approach could be used to develop energy efficient wireless communication systems, using less energy at emission of base stations or Wi-Fi boxes than now, creating much less electromagnetic pollution while still allowing the same quality of service. Here we mention that the electrical consumption of our SMM can be extremely small, as we show it in the Supporting Information, consistent with the fact that they could be used for green communications.

## Conclusion

In this Article we have asserted that tunable metasurfaces based SMMs alongside a simple energy feedback, permit to shape microwave complex fields resulting from multiply reverberated waves in a typical office room. It can be used to maximize the transmission between two antennas, hence focusing the waves onto well designed λ/2 isotropic focal spots or on the contrary to minimize the electromagnetic field on an antenna placed at any location in a room. We believe that this concept may find applications for indoor and urban wireless communications quality enhancements as we proved that the transmission between two antennas (possibly a mobile phone and a base station antenna) can be significantly increased. Another idea would be to use spatial microwave modulators in their minimization mode to provide an electromagnetic protection of small volumes. Interestingly, there is no need for large SMM to provide significant gains (or decrease the field to the noise level), as we performed our experiments using a small 0.4 m^2^ wall in a 66 m^2^ of total surface typical office room, and obtained relatively good results. Similarly, as those wall covers are very thin, we can imagine that they can be easily placed on furniture or walls. Although we focused in this study on a single polarization and a single frequency, multiple polarization and frequencies could be treated at once using a metasurface made out of various types of cross-polarized and different resonant frequencies subwavelength resonators, many of which are already available in the literature^8^,^9^,^10^,^11^,^12^. Finally we would like to add that the concept of SMM proposed here can be a very useful tool for the fundamental study of wave propagation in reverberating or through multiple scattering media.

## Methods

### Unit cell design

The unit cell (see Fig. S1(a)) is a dimer resonant element composed of two different parts: the resonant patch reflector (black in Fig. S1b) whose resonant dimension is L = 31 mm and its width is W = 45 mm and a parasitic strip (red in Fig. S1(b)) that is placed at a distance d = 0.6 mm away of the main reflector. All the electronics is concentrated on the parasitic strip to keep the resonance of the main reflector as unperturbed as possible. The strip is composed of two arms, linked by a pin diode (Infineon BAR 63-02V) which is biased with a voltage V (0 V or 5 V). Two choke inductors of 18 nH (TE Connectivity 36401J18NGTDF) are used to decouple the radio frequency from the DC bias voltage and avoid parasitic effects. Moreover, a 1.6 kΩ resistance is added to decrease the bias current to fit the diode's operating current. The cathode of the diode is grounded, so that, when the voltage V is set to zero, the diode blocks the current and only the first arm of the strip contributes to the strip's resonance. Conversely, when V = 5 V, the diode is forward-biased and the whole strip sets the resonance. In the first case, the so called π-state, the resonance of the parasitic strip (set by its dimensions) is *f_par_* much greater than *f_ref_*, the resonance frequency of the main reflector patch. Hence, the two resonant elements, the reflector and the parasitic strip do not hybridize, and the eigenfrequencies of the dimer are *f_ref_* and *f_par_*. In the second case, the so called 0-state, the resonance frequency of the parasitic strip is *f_ref_* so that it strongly hybridizes with the main reflector. The eigenfrequencies of the dimer are now *f_+_* and *f_−_*, respectively above and under *f_ref_*. This concept is summed up in Fig. 1(a) of the paper. The operating frequency *f_0_*, that is the frequency for which we want to optimize (or minimize) the electromagnetic field is chosen to fit *f_ref_*, so that the whole unit cell (the dimer) is either resonant (π-state, the field is reflected with a π phase shift) or transparent (0-state, the field is reflected without phase-shift). The patches are fabricated by classical etching of a copper layer (35 μm) on low loss substrate (NELTEC NH9338ST, tangent loss δ = 3e-3, permittivity ε = 3.4, height h = 1.5 mm).

### Near field measurements

The near field measurements of the resonant properties of all 102 patches (Fig. 1(a) and right inset of Fig. 1(c)) are implemented via two home-made near field probes. These are connected to a Network Analyzer (Agilent Technologies N5230A) and we focused on the transmission coefficient (S_12_). Both probes were positioned in the main reflector, each one at an edge of the resonating length L (to principally measure the resonance of the polarization along L and not along W). All patches were measured with both bias voltages 0 V and 5 V.

### Experimental set-up

Fig. S4 describes the experimental setup. The source antenna (S), a Wi-Fi vertically polarized monopole antenna (RADIALL R125.705.000W) is connected to one port of the network analyzer. The second port of the Analyzer is connected to the receiver antenna (R) which is the same type as (S) when the experiment consists in a simple optimization (or minimization) and is a non perturbative electro optical-probe (EFS-105 by ENPROBE Gmbh) when a spatial scan is realized. An amplifier (Amplical AMP0.7G4.2.30.29) is used to increase the Signal to Noise Ratio (SNR) when the electro-optic probe is used. Antenna (S) is mounted on a 400 mm long non-metallic arm, itself mounted on a rotating motor (Zaber TRS60C) which is used to achieve several configurations of disorder of the electromagnetic field within the room. (R) is mounted on a 2D translational stage (Newport M-IMS400PP) to scan the vertical polarization of the electric field in a 2D horizontal plane before and after an optimisation process. The scanning plane (as well as the optimization point) is chosen to lie around the half height of the metasurfaces to get the best control over the field. The 2D translational stage, that is partially metallic, is shielded with aluminium sheets to prevent the displacement of the antenna from perturbing the EM-field of the room. Each element of the reconfigurable metasurface is individually controlled (i.e. turned on and off) by one of the two single-board microcontrollers with each 54 digital output ports (Arduino Mega 2560). The same computer controls, through Matlab interfaces, the Network Analyzer, three motors (one rotative and two translational) and the two Arduinos. Finally, the windows of the room are partially shielded with aluminium to isolate the latter from external perturbations due to human displacements that strongly modify the EM-field.

### Realization of disorder

The probe is mounted on a 400 mm arm of a rotating motor Zaber TRS60C. When the motor is turned by 12° the antenna is moved by 84 mm which is superior to the correlation length of the field which is around 60 mm, as the wavelength at 2.5 GHz is around 120 mm. Hence, for each position of the source antenna (every 12°), we have another realization of the disorder in the room because the field is quite well uncorrelated to any previous one. This process is equivalent to changing completely and randomly the room itself though it is much simpler.

## Author Contributions

M.F. and G.L. developed the original concept. G.L. designed the SMM and experiments, theoretical model mentioned by mistake here and supervised the project. N.K. and M.D. fabricated and tested the SMM, and realized the experiments. N.K., M.D. and G.L. analyzed the results. N.K., M.D., G.L. and M.F. discussed the results and wrote the manuscript.

## Supplementary Material

Supplementary Information SI
