# Supplementary material for: Shaping complex microwave fields in reverberating media with binary tunable metasurfaces
Source: Sci Rep. 2014 Oct 21;4:6693. doi: 10.1038/srep06693 (PMC4204066; doi:10.1038/srep06693)
Supplement: Supplementary Information — SI [file srep06693-s1.pdf]

Shaping complex microwave fields in reverberating media with binary tunable metasurfaces

Nadège Kaina†, Matthieu Dupré†, Geoffroy Lerosey* and Mathias Fink*
Institut Langevin, ESPCI ParisTech and CNRS UMR 7587
1 rue Jussieu, 75005 Paris

†both authors contributed equally
*geoffroy.lerosey@espci.fr, mathias.fink@espci.fr

Supporting Information

1. The unit cell

a. Unit cell design

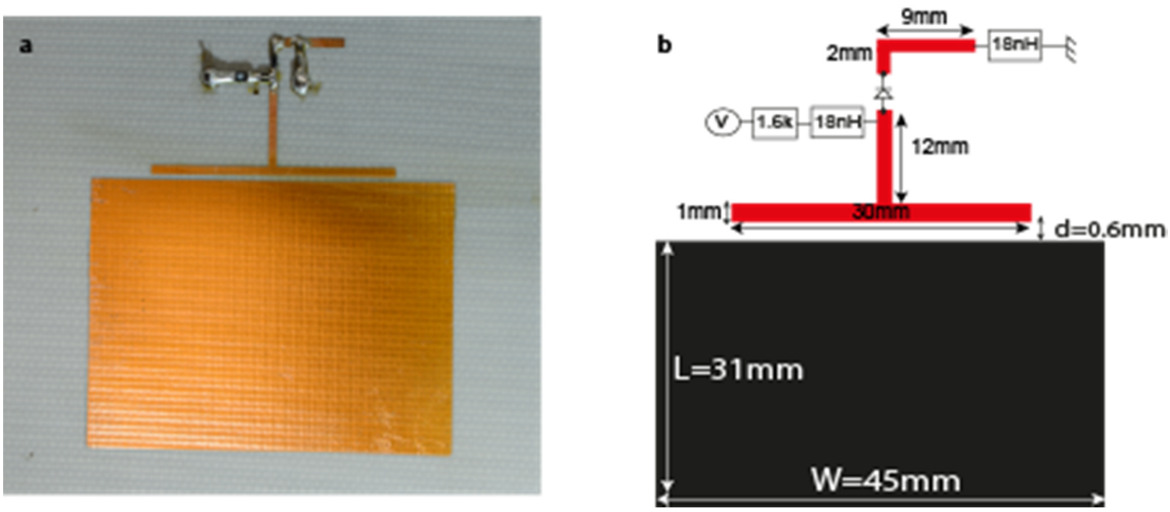

Figure S1. Photography and complete schematic view of the unit cell

In Supplementary Fig.1.A is presented the photograph of one resonant unit cell, that is composed of the resonant patch reflector (black in scheme), whose resonant dimension is L=31mm and its width is W=45mm. The parasitic strip (red in scheme) whose dimensions are given in Figure S1b is a distance d=0.6mm away from the patch reflector. All the electronics is concentrated on the strip to perturb the resonance of the main reflector as little as possible. The strip is composed of two arms, linked by a pin diode (Infineon BAR 63-02V) which is

biased with a voltage V (0V or 5V). Two choke inductors 18 nH (TE Connectivity 36401J18NGTDF) are used to decouple the radio frequency from the DC bias voltage and avoid parasitic effects. Moreover, a 1.6kΩ resistor is added to decrease the bias current to fit the diode's operating current. The cathode of the diode isgrounded, so that, when the voltage V is set to zero, the diode blocks the current and only the first arm of the strip contributes to the strip's resonance. Conversely, when V=5V, the diode is forward-biased and the whole strip sets the resonance. In the first case, the so called π-state, the resonance of the parasitic strip (set by its dimensions) is f_1 much greater than f_0, the resonance frequency of the main reflector patch. Hence, the two resonant elements, the reflector and the parasitic strip do not hybridize, and the eigenfrequencies of the dimer (strip + reflector = unit cell) are f_0 and f_1. In the second case, the so called 0-state, the resonance frequency of the parasitic strip is f_0 so that it strongly hybridizes with the main reflector. The eigenfrequencies of the dimer are now f_+ and f_-, respectively above and under f_0. This concept is summed up in the Figure 1.A of the main paper.

The operating frequency, that is the frequency for which we want to optimize (or minimize) the electromagnetic field is chosen to fit f_0, so that the whole unit cell (the dimer) is either resonant (π-state, the field is reflected with a ππ phase shift) or transparent (0-state, the field is reflected without phase-shift).

The patches are fabricated by classical etching of a copper layer (35µm) on low loss substrate (NELTEC NH9338ST, tangent loss δ=3e-3, permittivity ε=3.4, height h=1.5mm).

b. Simulations

We simulate using CST Microwave Studio an infinite array of patch resonators siting on a perfect ground plane and separated from it by a substrate that models the NELTEC used in experiments tangent (loss δ=3e-3, permittivity ε =3.4, height h=1.5mm). To do so, perfect electric boundaries are used in the direction perpendicular to the patch polarization (along W), and perfect magnetic conductor boundaries are used along the polarization of the resonator (along L). A port which emits a plane wave is created on the whole unit cell, two wavelengths away from the resonator, and it is used to measure the simulated S11, that is, the reflection coefficient from the infinite array.

We first start with a simple patch resonator that is simply connected to the ground plane through a diode (Fig. S2.A). The diode is modelled using the equivalent circuit given by the constructor, that is, a serial RLC with a 1.5Ω resistor and a 0.6nH inductor in the forward biased (0-state), and a parallel RLC with a 200fF capacitor and a 5kΩ resistor when the diode is reversed biased (π-state). In the π-state, the infinite array of reflectors presents a reflection dip of about -5dB at 2.45 GHz, corresponding to the losses in the metallic patch and in the substrate at the resonance frequency of the reflectors (Fig. S2B). Similarly, the phase profile (Fig S2.C) presents a 2π phase shift around the resonance, and it is of π exactly at the resonance, as was already widely studied in the literature *(21)*. When the diode is switched to the reversed bias mode, that is, in the 0-state, the resonance frequency of the patch is shifted to higher frequencies since the electrical length of the patch is lowered. A -20 dB dip is observed around 2.7 GHz, corresponding to the new resonance (Fig. S2.B).The latter is much deeper since on this state the small resistance of the diode absorbs a lot of the incident energy. Conversely, the observed phase shift around the resonance is much smaller, since most of the reflection comes now from the ground plane and not from the reflectors which present too much loss. It is clear that this type of reflector, used at 2.45 GHz would give us the expected features: a π phase shift in the π-state and a 0 one in the 0-state.

Nevertheless the diode capacitance is not a fixed value but it can vary quite a lot from a diode to another. Moreover the soldering and the electrical components used for the diode alimentation, presented in Fig. S1, add another non negligible complex impedance to the reflector, which is random and significantly shifts the resonance frequency of the patch. In Fig S2.d, we have simulated an infinite array of reflectors in the π-state while varying the parallel RLC capacitance around values typically available for the diode utilized. Clearly the resonance frequency of the π-state varies over a 50 MHz interval, meaning that the phase shift is strongly modified for each reflector array at 2.45 GHz, since this phase shift is quite steep. We do not want such a broad distribution of resonance frequency for the reflectors of the metasurface since it would considerably lower its influence on the multiply scattered waves at the working frequency. This effect would be even worse taking into account the electrical components and the manual soldering. Therefore we opt for a different design which, as we prove it here, is much less affected by the diode characteristics and its alimentation.

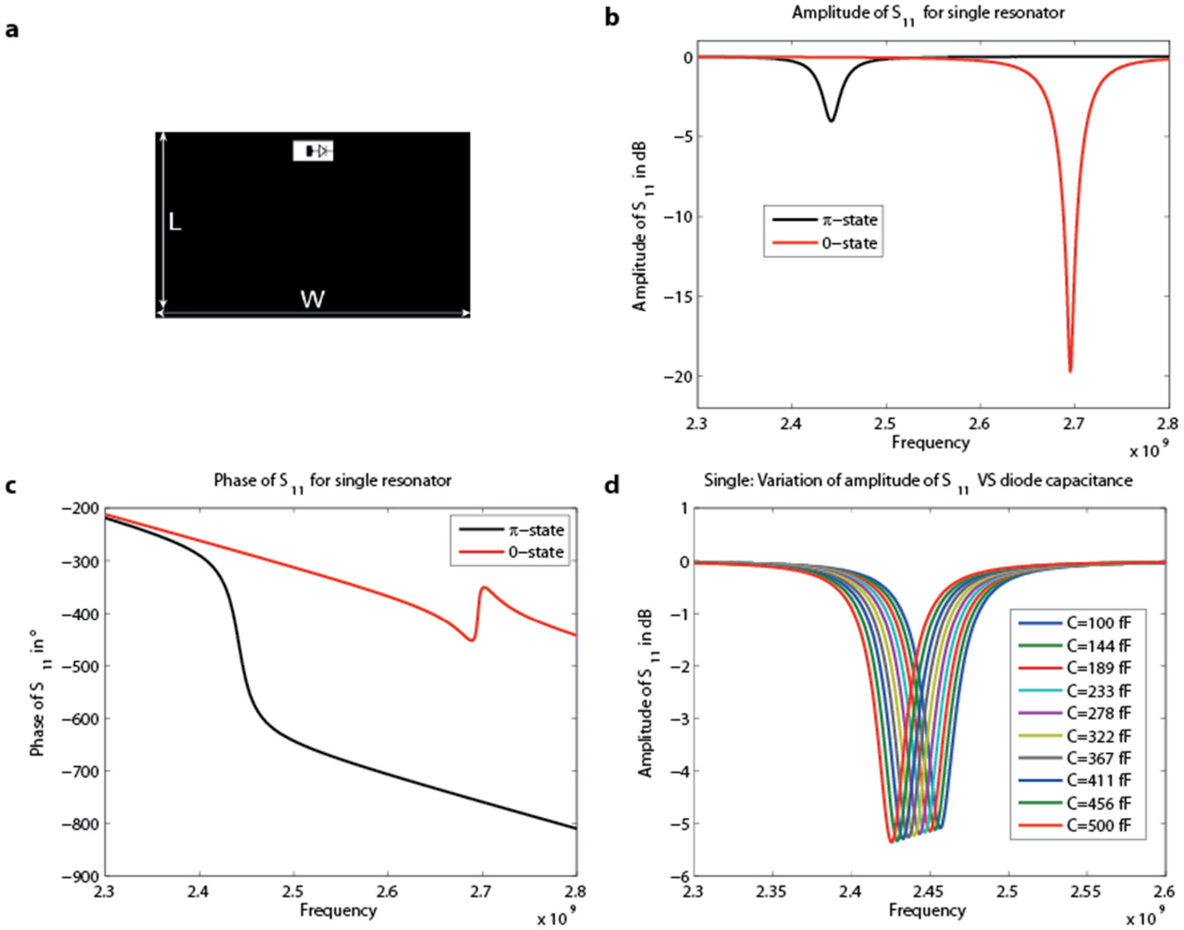

Figure S2. A single resonator patch reflector initially studied, B and C reflection coefficient (S_{11}, amplitude and phase) of an infinite array of single patch reflector, D variation of the reflection coefficient of infinite array of single reflectors as a function of the diode capacitance

To do so we work with the hybridized reflector described in the core of the paper and the unit cell description proposed here. In this design, the diode and the electrical components mostly affect the parasitic reflector. We scheme the reflector in Fig S3.a and we plot in Figs S3.b and c the phase and amplitude of the reflection coefficient of an infinite array simulated like the previous one using CST Microwave Studio. As expected, in the

π-state (diode reversed biased) the parasitic reflector does not influence the main reflector patch and we observe both a small -4 dB dip (corresponding again to losses in the substrate mostly) and a 2π phase shift around 2.45 GHz, the latter being exactly π at 2.45 GHz. In the 0-state, on the contrary, the two resonators strongly hybridize and a dimer is created with two resonance frequencies at 2.37 GHz and 2.55 GHz. The corresponding phase shifts are much smaller at those frequencies due to the energy absorbed by the small resistor of the diode in the forward biased mode (the reflection comes mostly from the ground plane, the dimer is poorly reflecting the waves). Cleary, in this state, the reflection of the infinite array at the working frequency is only given by the ground plane, leading to a 0 phase shift again. Now the interest of this design over the last one is clearly observable in Figure S3.D, where we again vary the capacitance of the diode in the reversed biased mode. We see dips around 2.45 GHz corresponding to the resonance frequencies of the simulated arrays in the π-state. Yet the latter are strongly concentrated on a 5 MHz bandwidth, due to the fact that we placed all the electronics on the parasitic resonator which in this state hardly influences the main reflector. Therefore the phase shift in the π-state is almost constant and so will it remain with imperfect soldering of the electronic parts. This is why we chose to work with this design for the tunable metasurface.

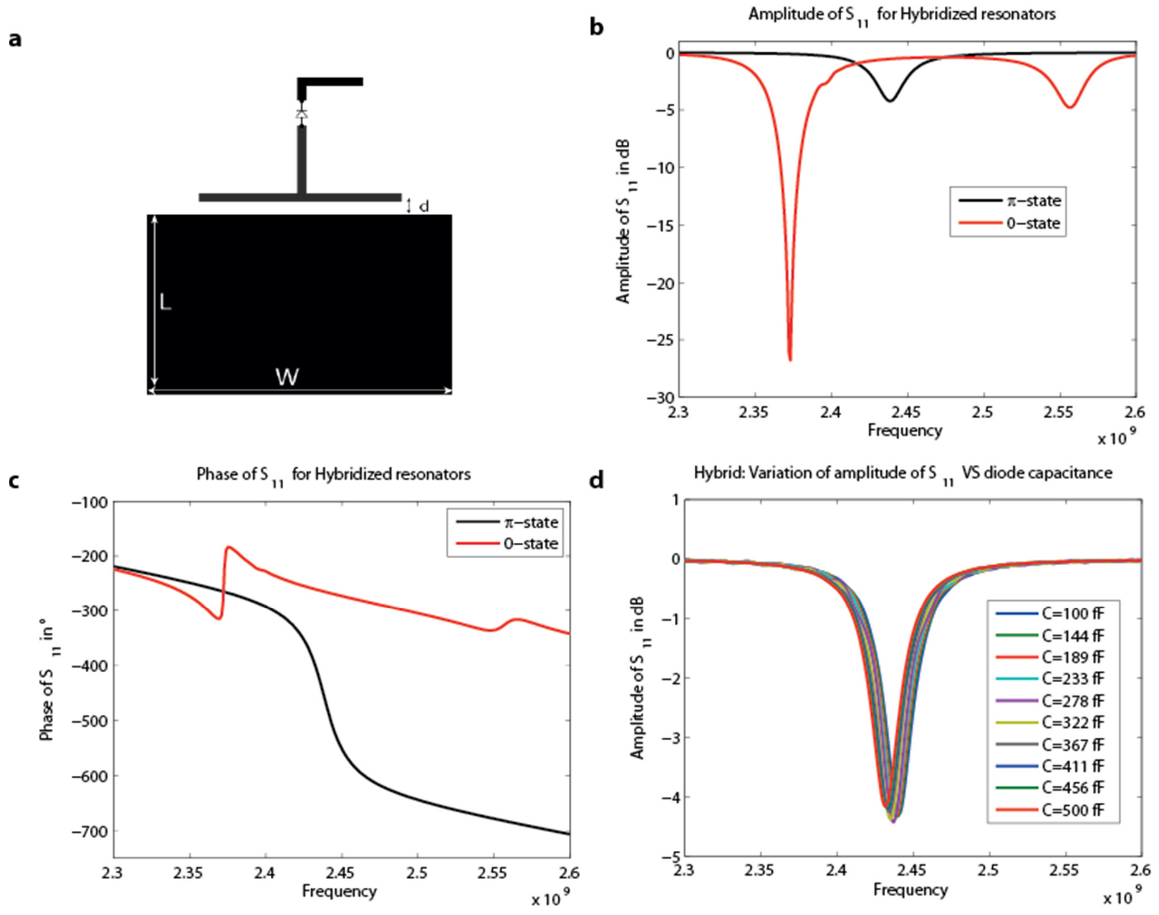

Figure S3. A, the hybridized resonators reflector used in the tunable metasurface, B and C, reflection coefficient (S_{11}, amplitude and phase) of an infinite array of hybridized patch reflector and D, variation of the reflection coefficient of infinite array of hybridized reflectors as a function of the diode capacitance.

c. Measurements (near field probes)

The near field measurements of the resonant properties of all 102 patches are implemented via two home-made near field probes. The latter are very short, hence very inefficient, electric wire so that the measurement is not perturbed by the environment. The two probes are connected to a Network Analyser (Agilent Technologies N5230A) and we focused on the transmission coefficient (S12). Both probes were positioned in the main reflector, each one at an edge of the resonating length L (to principally measure the resonance of the polarization along L and not along W). All patches were measured with the bias voltage 0V and 5V.

2. Experimental set-up

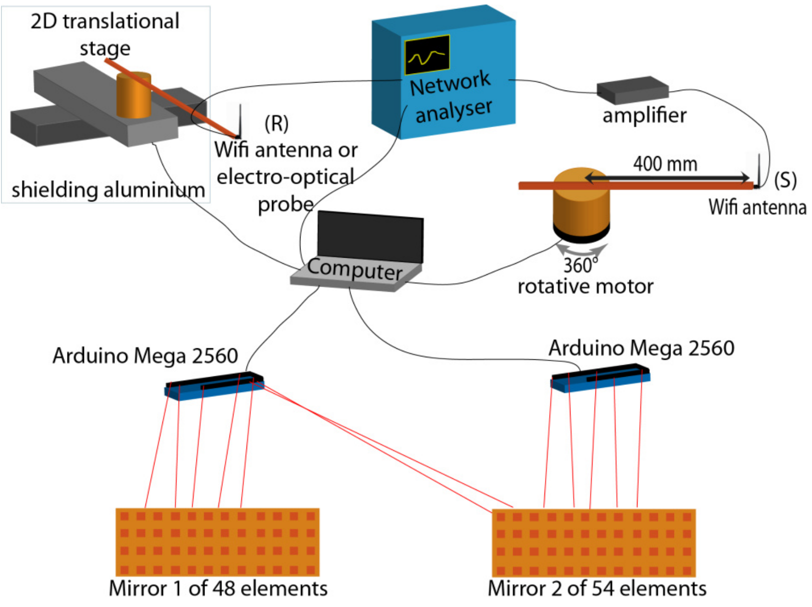

Figure S4. Schematic view of the experimental set-up

Figure S4 describes the experimental setup. The source antenna (S), a WIFI monopole vertically polarized antenna (RADIALL R125.705.000W) is connected to one port of the Network Analyser. An amplifier (Amplical AMP0.7G4.2.30.29) is used to increase the Signal to Noise Ratio (SNR) when the electro-optic probe is used. The second port of the Analyser is connected to the receiver antenna (R) which is the same type as (S) when the experiment consist of a simple optimization (or minimization) and is a non perturbative electro optical-probe (EFS-105 by ENPROBE Gmbh) when a spatial scan is realized. The S_{21} coefficient between (S) and (R) is measured by the Network Analyser.

Antenna (S) is mounted on a 400 mm long non-metallic arm, itself mounted on a rotating motor which is used to achieve several configurations of disorder of the electromagnetic field within the room (see paragraph.3). (R) is mounted on a 2D translational stage (M-IMS400PP) to scan the vertical polarization of the electric field in a 2D horizontal plane before and after an optimisation process. The scanning plane (as well as the optimization point) is chosen to lie around the half height of the metasurfaces, to get the best control over the field. The 2D translational stage, that is partially metallic, is shielded with aluminium sheets to prevent the displacement of the antenna from perturbing the EM-field of the room.

Each element of the reconfigurable metasurface is individually controlled (i.e. turned on and off) by one of the two single-board microcontrollers with each 54 digital output ports (Arduino Mega 2560).

The same computer controls, through Matlab interfaces, the Network Analyser, three motors (one rotative and two translational) and the two Arduinos.

Finally, the windows of the room are partially shielded with aluminium to isolate the latter from external perturbations due to human movements that strongly modifies the EM-field. This, of course, wouldn't be necessary in real application since the latter would be real time.

3. Realization of disorder

An ordinary furnished room behaves as a disordered cavity with a low Q factor for telecommunications and WIFI frequencies (600-2.5 GHz). Hence different modes of the room are excited when a telecommunication device is introduced. The spectrum of the transmission between two antennas in that room, or in other words the propagator is radically changed when one of those two antennas is moved from a distance greater than the correlation length of the field. This distance is, by definition, the distance we have to move an antenna to completely change the value of the field on that antenna. It is typically of the order of λ/2. As shown in Figure S5, the probe is mounted on a 400 mm arm of a rotating motor. When the motor is turned of 12° the antenna is moved of 84 mm which is superior to the correlation length of the field which is around 60 mm, as the wavelength at 2.5 GHz is around 120 mm. Hence, for every position of the source antenna every 12°, we have another realization of the disorder in the room because the field is quite well uncorrelated to any previous one. This is how the realization of the disorder is modified to perform several optimization processes in the same room. This process is equivalent to changing completely and randomly the room itself but it is much simpler.

Simulations of the enhancement

a. Modelling

In the optic domain, different algorithms have been proposed for optimizations or measurements with Spatial Light Modulators (SLMs) (13, 27, 29). In our case however, the amount of controllable channels of the room is much lower. A channel can be seen as a path that transmits a signal from a source S to a receiver R. If the source and the receptor are isotropic antennas that emit energy in all directions of the room, the number of available channels N in the room is equal to the number of independent paths in the room. As the walls of the room behave as mirrors with a reflection coefficient around 0.8, the number of channels is equal to the number of elementary areas of the room. Hence the number of channels, neglecting the windows, is given by: $N = \left(\frac{S}{\lambda/2}\right)^2$ where λ is the wavelength.

As in scattering matrix theory the room can be modelled by a matrix M of N by N complex random elements. For our rather small experimental room of 3 per 3 per 4 m^3 (that sets S to 66m^2 for the 6 faces of the room) and considering $\left(\frac{\lambda}{2}\right)^2 = 36$ cm² the surface of one channel, we have $N \approx 1.8\ 10^4$ channels. A source antenna (S) in the room is going to emit a signal through those N channels towards a receiver antenna (R), where the fields

emitted by (S) and received by (R) are N elements vectors. Therefore this can be mathematically modelled by the operation R=MS. Our metasurface is made of a small number n=102 of elements of area $(\lambda/2)^2$. That means that we can control only n=102 over the 18000 channels of the room. As we want to focus energy in only one channel (we optimize/minimize the field in one point that is part of one elementary area), we can consider that M is a 1 per N elements matrix, that R is a vector of only one element, and that we can π phase shift the first n=102 elements of M. At a given time t, we have:

$$R(t) = (m_1(t) \dots m_n(t)\ m_{n+1} \dots m_N) \begin{pmatrix} s_1 \\ \vdots \\ s_n \\ s_{n+1} \\ \vdots \\ s_N \end{pmatrix} \quad (1)$$

We define the enhancement of our optimization as: $\eta(t) = \frac{I(t)}{I(0)} = \left|\frac{R(t)}{R(0)}\right|^2$, and the averaged enhancement by: $<\eta> = \frac{<I(t)>}{<I(0)>} = \frac{<|R(t)|^2>}{<|R(0)|^2>}$.

The scheme of the algorithm is quite simple. At each step, one or several pixel of M is chosen, and π-shifted. Equivalently we can say that it is multiplied by -1. If the value |R| is greater than before, the change is accepted and we proceed to the next step of the algorithm. Otherwise, if |R| is lower than before, the pixel is not shifted. Two algorithms have been considered: a sequential algorithm, for which pixel are chosen one at the time from pixel number 1 to pixel number n, and partitioning algorithm where half of the pixels is chosen before being phase shifted, either randomly or accordingly to a Hadamard basis. The main advantage of the partitioning algorithm is that it is much less sensible to noise as the SNR is proportional to the number of shifted elements (n/2). However, in our case, the number of elements is quite small compared to the number of SLM elements in optics, and the sequential algorithm performs very well. Hence, the simulations of the partitioning algorithm are not presented here.

b. Influence of the size of the metasurface vs the size of the room

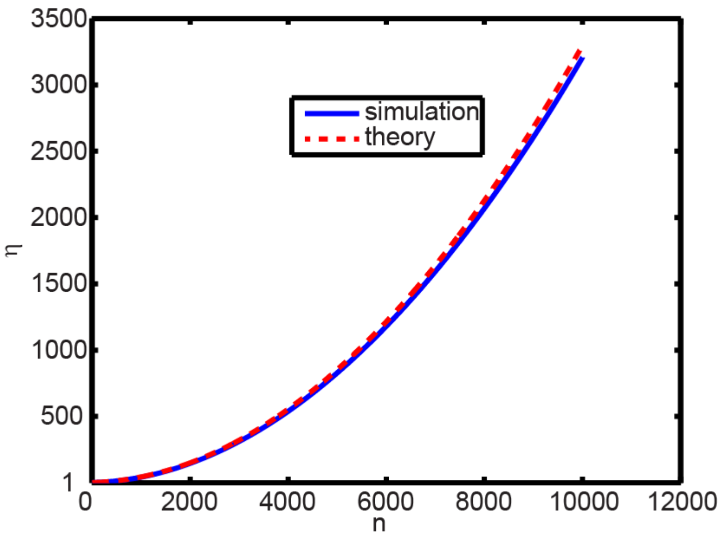

Figure S5. Simulated and theoretical enhancement as a function of n for N=10.000 and without any noise.

Noise is the most obvious phenomenon responsible of decreasing η compared to an ideal case, but it is not the only one. The decorelation time of the medium (the room) compared to the time of an optimization is important too. However, in our experiments, nothing moves in the room and we can consider that the decorelation time is infinite. Moreover, it would be easy to get real time optimizations with some slight modifications of the setup such as using an antenna and a receiving unit instead of a network analyser. The third, and probably the most important criterion that influences η is the ratio of the size of the mirror compared to the size of the room. As previously explained, we can consider that we control only 102 channels over the 18000 channels of the room but this corresponds to the worst possible case for which no antenna faces the metasurface. If an antenna is placed in the line of sight of the metasurface, the effective number of controlled channels in the room is increased. Those uncontrollable channels introduce a bias signal that adds to the S_{21} signal on the antenna. Hence the measured signal can be seen as the sum of three signals: $S_{21}=S_{mirror}+S_{bias}+S_{noise}$.

Of course, only S_{mirror} can be optimized. Noise has to be studied apart and is neglected here. Initially, all channels of the room add randomly and the total amplitude is of the order of $\sqrt{N}$. However, the sequential algorithm tries to add coherently the n channels of the mirror, and the signal after optimization is finally proportional to $n+\sqrt{N-n}$. Finally, the approximated amplitude enhancement is after optimization:

$$< \eta > \propto \left(\sqrt{1-\frac{n}{N}}+\frac{n}{\sqrt{N}}\right)^2 \simeq (1+\frac{n}{N})^2 \qquad (2)$$

A more detailed calculation gives the exact coefficient for η as given in equation (4). A noticeable fact is that for large value of N>>n, the enhancement is proportional no n^2 contrary to optical studies (28), for which n=N, and that have shown that the enhancement is proportional to n, which is in agreement with our previous formula.

Fig.S5. shows the simulated and theoretical enhancements as a function of the number of elements of the metasurface for a room of 10.000 elements and in the absence of noise. Theoretical enhancement is plotted according to equation (4) given in in §5.a. Both are in agreement and show an enhancement proportional to n^2. This can seem unexpected compared to previous optical studies *(18,28)*, and that display an enhancement proportional to n which is equal to N. However this is only due to the fact that here, we have a total number of channels that is fixed. Only the number of controllable channel is optimized. Moreover, n and N are calculated for the parameters of the cavities in the main text.

c. Comparison with experiments

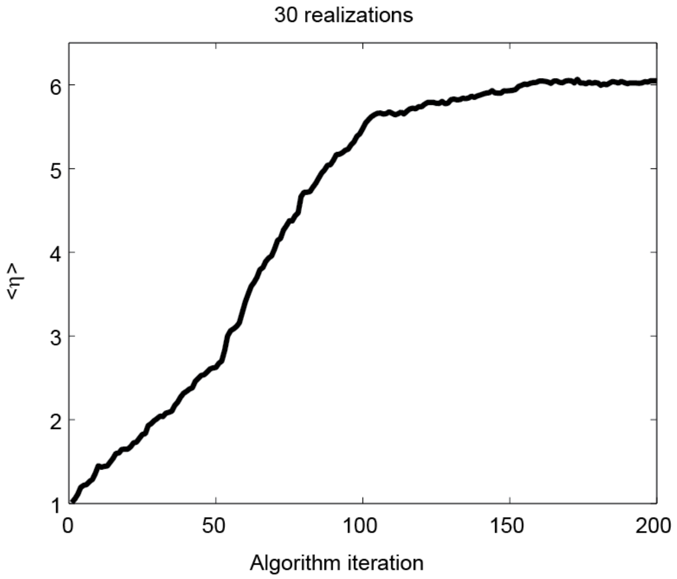

Figure S6. Experimental linear optimization, averaged over 30 realizations

From Fig.S6. We can extract the values corresponding to our experimental conditions for which n=102. The corresponding enhancement for n=102 is η=2.3. Quantitatively, the simulated enhancement is lower than the experimental one (η=6) when the receptor is placed in the line of sight of the metasurface. However for out of sight experiments the averaged experimental enhancement is of the order of magnitude of the simulated one. In both experiments and simulation the maximum enhancement is achieved for a number of iterations of the algorithm equal to n.

4. Binary spatial microwave modulator

a. Influence of the binary only modulation

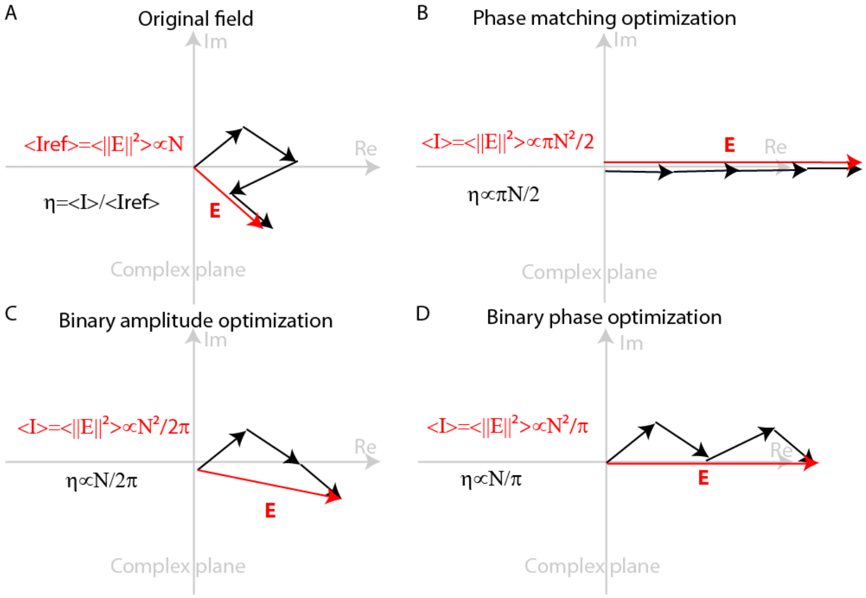

Figure S7. Scheme of the optimization process, for different type of optimizations. A: Orignal electric field before optimization. B: Field obtained ofter a perfect phase optimization. C: Field obtained after a binary amplitude optimization. D: field obtained after a binary phase optimization.

Here, we consider the effect of using a binary only phase modulation instead of a perfect phase modulation. Fig.S7 shows the scheme of a different optimisation process. In a room the field (red vector of Fig.S7.) is the result of the sum of the fields transmitted through a great number of channels N (black vectors in Fig.S7). A priori they add destructively in the complex plane to give an averaged intensity proportional to N (Fig.S7.A). If we perform a phase matching modulations (Fig.S7.B), we align all those vectors on the real axis to get an intensity proportional to N^2. On the other hand, if we perform a binary amplitude modulation (Fig.S7.c), we only conserve the vectors which have a positive real part, while others (in average, half of the total number of vectors) are set to zero *(30)*. The enhancement is again proportional to N^2 but with a reduced coefficient of $1/(2\pi)$ instead of $\pi/2$. Now if we perform a binary phase modulation (Fig.S7.d), we keep the vectors with a positive real part, while the others are multiplied by -1 so that they now have a positive real part. In that case, the Intensity is twice the one obtained with a binary amplitude modulation because those vectors (in average, half of the total number of vectors) are conserved instead of being cancelled. As shown in *(30)*, for a binary amplitude optimisation the enhancement would be for great values of n and N compared to 1:

$$< \eta > \simeq 1 + \frac{1}{2\pi}\frac{n^2}{N} \qquad (3)$$

Hence for a binary phase optimisation we expect the exact coefficient to be $1/\pi$ instead of $1/2\pi$. The exact formula for η is then:

$$< \eta > \simeq \left(1 + \frac{n}{\pi N}\right)^2 \qquad (4)$$

b. Correlation of the field on SMM

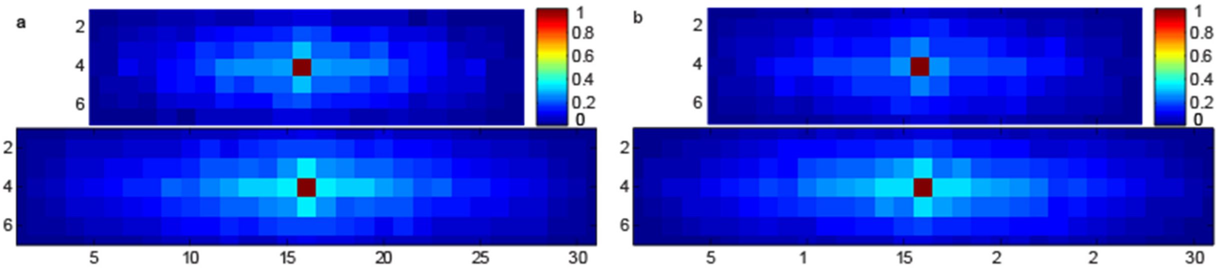

Figure S8. Autocorrelations of the final masks of the optimizations for the two parts of the SMM. A: Average on 30 maximizations. B: Average on 30 minimizations.

A question that we may raise is if the field on a wall of a room in general, and especially on the metasurface, is really random, that is, if its correlation length is exactly half a wavelength or much larger. The state of the metasurface can provide an answer. Indeed, after optimization, it gives a binary estimation of the phase of the field on the metasurface. For instance, if after an optimization all the elements of the metasurface are in the same state, and knowing that they interfere constructively on the receptor, we can deduce that we have a plane wave incident on the metasurface. And if after an optimization all elements take the opposite value of its nearest neighbors, knowing that they interfere destructively on the receptor, we can again deduce that we have again a plane wave on the wall. On the two dimensional autocorrelation of the states of the metasurface, we would see this effect: the distance of the correlation would be larger than λ/2. On the contrary, if the incident field on the metasurface is random, all elements randomly take the value 0 or 1, and the distance of correlation is of the order of the λ/2. This is exactly what we can see on Fig.S8.: the half peak width of the maximum of the autocorrelation is one element large, which is designed at λ/2. Hence we can indeed consider that we have a random electromagnetic field on the walls of a furnished room with a coherence length of about half a wavelength.

5. Out of sight experiments

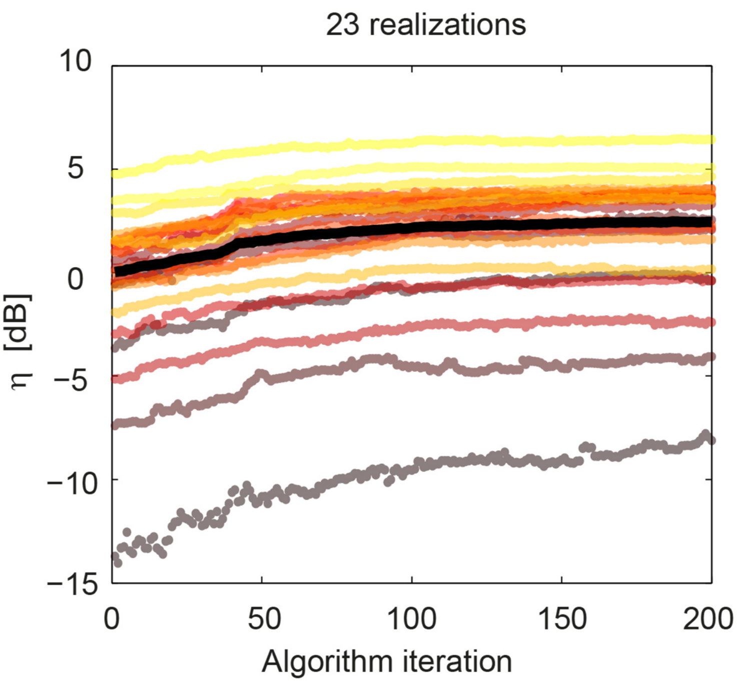

Figure S9. Experimental averaged optimization (over 23 realization of disorder) on a receiver antenna (R) placed out of sight of the both the metasurface and the source antenna (S), all parts being spaced by 3 meters.

From Figure S9 we show that even in the worst possible configuration for the receiver antenna (a monopole that is not placed in the direct sight of the metasurface), we achieve some enhancement if the field amplitude on the antenna. The averaged enhancement is 2.5 dB, with a standard deviation that can approach 1.5 dB in the end of the optimization process. The maximum gain measured, for the lowest initial intensity reaches 6.5 dB. This result proves that changes on the metasurface can impact the whole volume of the room, even if it is subsequently less efficient.

6. Power Consumption

Now if our approach is to be used to design greener wireless communications in the future, the SMMs have to be very energy efficient. We hence have to evaluate the energy consumption of the developed SMM, and compared it to the benefits in terms of energy. For our walls, the electric consumption can be divided in two parts: the pixel and the micro-controller consumptions. The latter is very weak since the boards which perform the optimizations and control the pixels are based on logic units termed picowatt devices by its designer, and consume less than a few mWs per hundreds of channels. Since our SMMs do not generate waves but simply reflect existing ones in a clever way, energy is only consumed in the feeding electrical circuit of the diode used to tune the pixel. These diodes are low power ones since the electromagnetic energy actually exciting the resonators is relatively low, and they are hence fed with a low voltage (5 V). The pixel consumption depends on the current that runs through the circuit, and it is set by the resistance R we put in the feeding circuit of the diode. In our initial design we used R = 1.6 kΩ and the measured consumed power is P = 1.4 mW per pixel, which is

quite high. This value can be significantly lowered by increasing the resistance. We have performed further experiments to check the consumption and characteristics of our designed reflector with increasing resistance. The results are shown in Fig. S10.*A-D*, in which we plot the measured amplitude and phase of microwaves reflected (S_{11}) by a single pixel in a single mode waveguide in the 0-state and the π–state. They prove that we can use up to a 300 kΩ resistance without altering significantly the properties of one pixel at the frequency of interest. This corresponds to a consumed power, as proved in Figure S10.E, of about 50 µW per feeding circuit. Moreover, it is to be reminded that this consumption is calculated for a pixel that is biased with 5 V (the 0-state). One pixel is however statistically as much set to 0 V (π-state) as to 5 V (0-state) so that the average consumption of one pixel is half of the calculated one. This means that with no special engineering of our system, each pixel consumes about 25 µW on average, which first proves that our SMMs are energy efficient, and also that they typically consume an electric energy amount similar to or lower than the amount of electromagnetic energy they actually recycle. Here, we state that this study was conducted as a complementary study once the metasurface of 102 elements was already fabricated and tested with the initial randomly chosen value of 1.6kΩ. That explains why the value of the resistance in the experiment is not the optimal one.

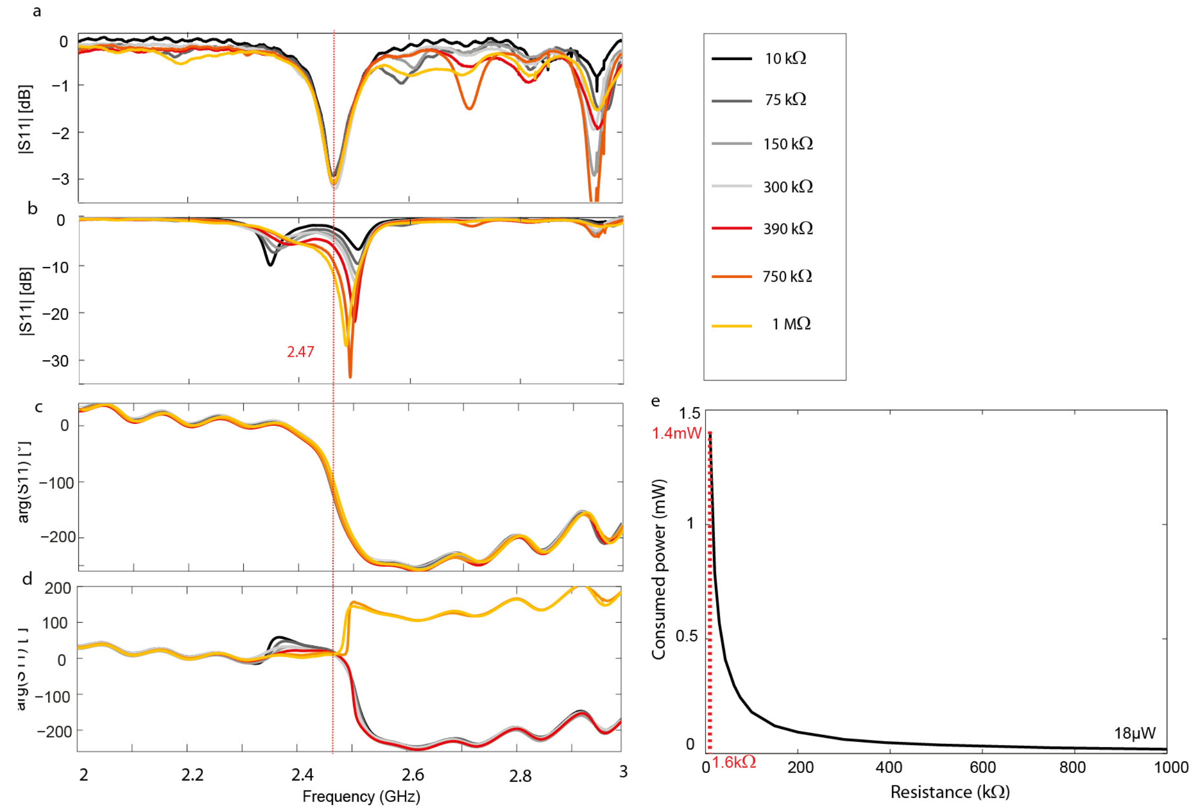

Fig.S10: Electric consumption (a-d) Measured reflection coefficient in amplitude and phase of one pixel for both 0-state (a-c) and π-state (b-d) when the resistance of the electric circuit is modified. (e) Measured electric consumption of one pixel when it is set to its 0-state mode (5 V bias).
